# The effect of HIV and antiretroviral therapy on characteristics of pulmonary tuberculosis in northern Malawi: a cross-sectional study

**DOI:** 10.1186/1471-2334-14-107

**Published:** 2014-02-25

**Authors:** Lumbani Munthali, Palwasha Y Khan, Nimrod J Mwaungulu, Femia Chilongo, Sian Floyd, Michael Kayange, Judith R Glynn, Neil French, Amelia C Crampin

**Affiliations:** 1 Karonga Prevention Study, Chilumba, Malawi; 2 London School of Hygiene and Tropical Medicine, London, UK; 3 Karonga District Hospital, Ministry of Health, Karonga, Malawi; 4 Institute of Infection & Global Health, University of Liverpool, Liverpool, UK

**Keywords:** Smear positivity, Cavitation, HIV, ART, Infectiousness, Culture, Malawi

## Abstract

**Background:**

HIV infection reduces the likelihood that individuals with pulmonary tuberculosis are smear positive and that they have cavitatory disease. Antiretroviral therapy (ART) may shift the pattern of disease to be more similar to that of HIV negative patients. This would aid diagnosis- which often depends on sputum smears – but would also increase infectiousness. We assessed the effect of HIV and ART on smear positivity and cavitatory disease in laboratory-confirmed pulmonary TB patients.

**Methods:**

Three sputum samples were collected per pulmonary TB suspect and were examined using microscopy and culture. Chest radiographs were available for a subset of patients as part of another study. The effect of HIV and ART status on sputum smear positivity and lung cavitation were evaluated using multivariable logistic regression.

**Results:**

Of 1024 laboratory-confirmed pulmonary TB patients who were identified between January 2005 and December 2011, 766 had HIV and ART status available. Positive sputum smears were significantly more common among HIV negative individuals than HIV positive individuals (adjusted OR = 2.91, 95% CI 1.53 – 5.55). Compared to those HIV positive but not on ART, patients on ART were more likely to be smear positive (adjusted OR = 2.33, 95% CI 1.01 – 5.39) if they had been on ART ≤ 6 months, but only slightly more likely to be smear positive (adjusted OR = 1.43, 95% CI 0.68 – 2.99) if they were on ART > 6 months. HIV negative patients were more likely than HIV positive patients to have cavitatory disease (adjusted OR = 1.97, 95% CI 1.20 – 3.23). Patients on ART > 6 months had a slight increase in cavitatory disease compared to HIV positive patients not on ART (adjusted OR = 1.68, CI 0.78 – 3.63).

**Conclusions:**

HIV infection is associated with less smear positivity and cavitation in pulmonary TB patients. Among HIV positive patients, the use of ART shifts the presentation of disease towards that seen in HIV-negative individuals, which facilitates diagnosis but which also could increase infectiousness.

## Background

Tuberculosis (TB) is a major public health challenge. Globally there were an estimated 8.7 million incident cases of TB in 2011, 26% of which occurred in Africa, and 1.4 million TB deaths [1]. 13% (1.1 million) of the incident cases were HIV positive. In Malawi the number of TB cases notified to the National TB Control Programme rose sharply from 12,395 (132 per 100,000 population) in 1990 to 25,491 (199 per 100,000 population) in 2005 [2]. The upsurge in TB was largely due to HIV infection. HIV increases the risk of rapid TB progression after primary infection or re-infection with *Mycobacterium tuberculosis (Mtb)* and increases the risk of reactivating latent TB infection [3,4]. In HIV negative individuals infected with *Mtb*, the lifetime risk of developing TB disease is between 10% and 20% while in those co-infected with HIV the annual risk is 10% or more [5,6].

The Malawi government, with assistance from the Global Fund on Tuberculosis, Malaria and HIV/AIDS, started provision of antiretroviral therapy (ART) to HIV-infected eligible individuals in 2004. By mid-2006 there were 94 facilities providing ART and the total number of patients alive and on ART was 41,549 [7]. The provision of ART has been widely scaled up including the introduction of “Option B+” in Malawi in July 2011 with which all HIV-infected pregnant and breastfeeding women are started on lifelong ART regardless of clinical or immunological stage. By the end of 2012 there were 651 static ART sites and 585 sites providing Option B+ and a total of 404,905 HIV-infected individuals on ART [8]. Since the introduction and rolling out of ART in Malawi TB notification rates have started to decline, with 25,491 (199 per 100,000 population) in 2005, 23,929 (171 per 100,000 population) in 2008, 22, 674 (157 per 100,000 population) in 2009 and 21,092 (142 per 100,000 population) in 2010 [2]. In HIV-infected individuals, ART reduces the incidence of TB, although rates remain higher than in HIV negative patients [9-11].

Sputum smear microscopy has been the backbone for the diagnosis of pulmonary TB in most low-income countries because of its low cost, and is directly related to infectiousness [12-14]. Due to paucibacillary pulmonary disease in patients infected with HIV, the use of smear microscopy in the diagnosis of pulmonary TB is not ideal, although widely used [14]. In HIV-infected, immunocompromised individuals, lung cavitation is less frequent because the defective specific cell-mediated immune response reduces the ability to mount a granulomatous response to *Mtb*[15,16].

ART is widely used and has significantly improved the prognosis of HIV-infected patients. The objective of this study is to assess the effect of HIV and ART on the presence of smear positivity and cavitation at diagnosis of laboratory-confirmed pulmonary TB, and to understand if ART may affect infectiousness of HIV positive TB patients.

## Methods

### Study setting

Karonga district is situated in northern Malawi and has a population of approximately 300,000 [17]. The district has a district hospital and several health centres. The Karonga Prevention Study has been conducting research on tuberculosis in the district since 1988. Pulmonary TB cases were identified through enhanced passive surveillance: individuals with a cough of at least two weeks visiting health facilities within the district were investigated for pulmonary TB. Individuals were also identified by community screening in the context of other related research studies, but this practice yielded few cases. Individuals suspected of having pulmonary TB were asked to submit three sputum specimens, one collected on-the- spot, a second early morning specimen and a third specimen collected on-the-spot at the time of delivery of the second specimen, as per Malawi National TB Programme guidelines [18,19].

### Laboratory methods

Sputum smear examination was performed at the research project laboratory using fluorescence microscopy with auramine staining, with confirmation of positive smears by light microscopy using Ziehl-Neelsen (ZN) stain. All sputum specimens were then cultured using Lowenstein- Jensen solid media [20]. Cultures suggestive of TB were sent to the National Mycobacterium Reference Laboratory (Health Protection Agency) in the UK for species confirmation and drug sensitivity testing.

### Case definition

A pulmonary TB case was defined as being laboratory confirmed if at least one sputum smear or culture was positive. Those with no positive culture and only a single scanty smear (<10 acid-fast bacilli per 100 High Powered Fields) were excluded.

### Study population, data collection and statistical methods

All confirmed pulmonary TB cases in Karonga District aged ≥15 years and diagnosed between January 2005 and December 2011 were included in the study. After written informed consent, study participants were interviewed using a standard structured questionnaire. In individuals aged <18 years a written informed consent was obtained from their parents or guardians at the time of the interview. Patients were asked about sociodemographic characteristics and history of previous TB.

Chest radiographs were available for a group of patients, most of whom were enrolled in immunological studies. The objective of these studies was to develop a rapid, sensitive, and affordable biological marker (genomic or proteomic) for diagnosis of TB. Inclusion criteria for immunological studies included: willingness to undergo an HIV test and give a venous blood sample, and aged 18 years or over. Exclusion criteria were a past history of cancer, taking steroids in the past 6 months, pregnancy and diabetes: few individuals were excluded for these reasons. Radiographs were independently reviewed by a chest physician or a radiologist following a standardised format that assessed the presence of cavitation, parenchymal consolidation, pleural effusion, miliary pattern infiltrates and other radiological abnormalities. These readers were blinded to the HIV and ART status of the patients. They were also blinded to the diagnosis of the patient, which was possible because they were also reviewing chest radiographs for individuals with illnesses other than TB.

Pulmonary TB patients were pre and post-test counselled for HIV by trained counsellors. After interview and if written consent was given blood was taken. In 2005 – 2007, HIV testing was done using ELISA (Vironostika; Organon Teknika, Cambridge, UK) and particle agglutination test (Edgeware modification of the Serodia). Positives were confirmed using a further ELISA (Wellcozyme Wellcome Diagnostics, Dartford, UK or Enzygnost, Behring, Marburg, Germany) and particle agglutination test assay (Serodia, Fujirebio Inc, Tokyo, Japan). Samples giving discrepant results were repeated in duplicates using the same two tests. From 2007, HIV testing was done using Determine (Abbot Japan Co, Tokyo, Japan). Positives were confirmed using Uni-Gold (Trinity Biotech plc, Bray, Ireland). For any discrepant results Bioline (Standard Diagnostics, Giheung-gu, Korea) was used. Patients who were known to be HIV positive at the time of TB diagnosis were asked about ART use. Free ART was available in the District from 2005. As well as patients’ self-reports, information on ART use was collected from patient-held “health passports” which record details of treatments. Further data on ART usage was also available from linked studies in local ART clinics, which involve identifying patients on ART, if they give consent.

Statistical analysis was performed using STATA 11© software (StataCorp, TX, USA). Patients were grouped as ‘not on ART’, ‘on ART ≤6 months’, ‘on ART >6 months’ or ‘HIV negative’. Duration on ART was included to differentiate the effect of prolonged improved immune function from the early period in which TB could result from unmasking or continued poor immunity [21]. The main analysis was restricted to pulmonary TB cases with known HIV status. The effects of HIV and ART status were evaluated using multivariable logistic regression with sputum smear positivity (among 766 study participants) and cavitation (among a subset of 310 study participants) as the outcome variables.

The data were derived from studies approved by the Malawi National Health Sciences Research Committee which is the local ethics committee and the Ethics Committee of the London School of Hygiene & Tropical Medicine.

## Results

### Characteristics of the study population

**Figure 1:**
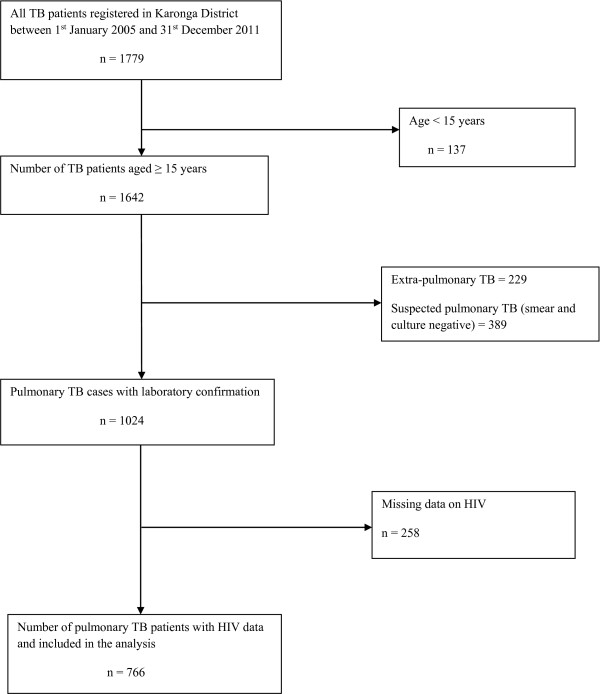
Flowchart showing pulmonary TB patients included in the analysis.

There were 1642 cases of TB aged ≥15 years registered in the 7 year period from January 2005 to December 2011. Of the 1642 TB cases, 1024 (62%) were pulmonary with laboratory confirmation, and of these, 766 (75%) had known HIV status and were included in the analysis (Figure 1). This included 689 (90%) who were culture-positive.

Those with unknown HIV status were older than patients with known HIV status but there was no evidence for other differences in terms of gender or socio-economic status. There were differences in age, sex and socio-economic characteristics between HIV negative and HIV positive patients. HIV negative patients were older, more commonly male, more likely to be farmers and to have received less education. Previous TB was more common in HIV positive patients, particularly those who had been on ART for more than 6 months.

Table 1 shows the prevalence of smear positivity and lung cavitation by patient characteristics among pulmonary TB cases. Of the 766 cases, 368 (48%) were female. The prevalence of HIV infection was high, with 475 (62%) of the 766 TB cases being HIV positive. Of the 475 patients with HIV infection 191 (40%) had initiated ART by the time they were diagnosed with TB.

**Table 1:** Prevalence of sputum smear positivity and cavitation by patient characteristics among pulmonary TB patients

	**Smear positive**	**Cavitation**
**Characteristic**	**n/N (%)**	**n/N (%)**
Overall	691/766 (90%)	158/310 (51%)
HIV status		
HIV positive	416/475 (88%)	72/164 (44%)
HIV negative	275/291 (95%)	86/146 (59%)
ART status		
HIV positive not on ART	242/284 (85%)	34/84 (40%)
HIV positive on ART 0 – 6 months	93/100 (93%)	16/39 (41%)
HIV positive on ART >6 months	81/91 (89%)	22/41 (54%)
History of previous TB		
No	606/668 (91%)	140/279 (50%)
Yes	82/91 (90%)	18/30 (60%)
Age (years)		
15 – 29	175/185 (95%)	47/81 (58%)
30 – 44	331/368 (90%)	68/142 (48%)
45 – 59	133/152 (88%)	31/59 (53%)
≥60	52/61 (85%)	12/28 (43%)
Sex		
Male	365/398 (92%)	92/175 (53%)
Female	326/368 (89%)	66/135 (49%)
Education		
Never or lower primary	187/206 (91%)	41/78 (53%)
Higher primary	306/339 (90%)	76/134 (57%)
Secondary	107/113 (95%)	25/47 (53%)
Tertiary	76/91 (84%)	13/44 (30%)
Occupation		
Farmer	349/387 (90%)	86/156 (55%)
Professional	60/67 (90%)	7/29 (24%)
Skilled manual	86/90 (96%)	20/41 (49%)
Unskilled	108/122 (89%)	26/46 (57%)
Small trader	68/78 (87%)	12/26 (46%)

Chest radiographs were available for 310 (40%) of the 766 patients. A higher proportion of HIV negative patients had chest radiographs, and these were slightly more common in men, but there was no evidence that having a chest radiograph was associated with age, or socio-economic status of the study participant.

### Smear positivity and cavitation

Overall, 691 (90%) of the 766 patients had at least one sputum smear positive for acid-fast bacilli, and 158 (51%) of the 310 patients with X-rays had evidence of cavitation (Table 1). The proportion smear positive was higher in HIV negative patients (95%) than in HIV positive patients (88%). Among those who were HIV positive, the proportion smear positive was highest for those on ART for less than 6 months (93%) and lowest in those not on ART (85%). Cavitation was more common in the HIV negative (59%) than in those who were HIV positive (44%). It was present in 54% of those on ART for >6 months and 40% of those not on ART.

Table 2 shows the association between HIV and ART status with sputum smear positivity and cavitation. After adjusting for age and sex the association between HIV status and smear positive pulmonary TB remained. HIV negativity was associated with nearly three times increase in the odds of smear positivity compared to HIV positive patients (adjusted OR = 2.91, 95% CI 1.53 – 5.55). HIV positive patients on ART ≤6 months had more than twice the odds of being smear positive compared to the HIV positive not on ART (adjusted OR = 2.33, 95% CI 1.01 – 5.39) while those on ART >6 months had nearly one and half times the odds of smear positivity (adjusted OR = 1.43, 95% CI 0.68 – 2.99).

**Table 2:** Association of HIV and ART status with sputum smear positivity (amongst 766 participants) and cavitation (amongst 310 participants)

**Outcomes**	**Smear positivity (n = 766)**				**Cavitation (n = 310)**			
	**Univariable analysis**		**Adjusted for age and sex**		**Univariable analysis**		**Adjusted for age and sex**	
**Characteristic**	**Crude OR (95% CI)**	**p-value**	**Adj OR (95% CI)**	**p-value**^**(a)**^	**Crude OR (95% CI)**	**p-value**	**Adj OR (95% CI)**	**p-value**^**(a)**^
HIV status								
HIV positive	1.00		1.00		1.00		1.00	
HIV negative	2.44 (1.37 – 4.32)	0.002	2.91 (1.53 – 5.55)	<0.001	1.83 (1.17 – 2.88)	0.009	1.97 (1.20 – 3.23)	0.007
ART status								
HIV positive not on ART	1.00		1.00		1.00		1.00	
HIV positive on ART 0 – 6 months	2.31 (1.00 – 5.31)	0.095	2.33 (1.01 – 5.39)	0.091	1.02 (0.47 – 2.22)	0.349	0.92 (0.41 – 2.03)	0.328
HIV positive on ART >6 months	1.41 (0.67 – 2.93)		1.43 (0.68 – 2.99)		1.70 (0.80 – 3.61)		1.68 (0.78 – 3.63)	

OR = Odds Ratio; Adj OR = Adjusted Odds Ratio; CI = Confidence Interval.

^(a)^Likelihood ratio test p-value.

After adjusting for age and sex, HIV negativity was associated with nearly twice the odds of cavitation of those who were HIV positive (adjusted OR = 1.97, 95% CI 1.20 – 3.23). HIV positive individuals who were on ART > 6 months had more than one and half times increase in the odds of cavitation as compared to HIV positive patients not on ART (adjusted OR = 1.68, CI 0.78 – 3.63). Adjustment for other possible confounders (history of TB, level of education and occupation) made little difference to the results for smear positivity or cavitation.

## Discussion

This study shows that smear positivity and radiological changes in laboratory confirmed TB patients diagnosed in a rural northern Malawian population were affected by the individual’s HIV and ART status. HIV-infected individuals were significantly less likely to have positive sputum smears and lung cavitation than those who were HIV negative, as found elsewhere [6,22]. ART appears to increase smear positivity and cavitation in HIV positive individuals although the proportions remained lower than in HIV negative individuals.

Many studies have looked at ART and its effect on survival in HIV-infected individuals [23,24]. Fewer studies have described the role of ART in the evaluation of people suspected of having TB [25]. Tiffany et al. found that among HIV positive TB patients in the US those who were diagnosed in the late ART era were more likely to have a positive smear compared to those diagnosed in the early ART era [26]. A study in Italy found that cavitation was more common in HIV infected TB patients who were on ART compared to those not on ART [27]. Sharma et al*.* in India showed that HIV positive individuals with a moderate functional immune system diagnosed with pulmonary TB had sputum smears that were more likely to be positive for acid fast bacilli than those with higher levels of immunosuppression [28].

The positive association between ART and smear positivity and cavitation suggests that ART may play a role in shifting the disease pattern towards a more typical appearance, similar to that found in HIV negative individuals due to restoration of immune function. An increasing proportion of HIV–infected TB patients are likely to present with a laboratory confirmed TB diagnosis or a typical radiographic appearance on a chest radiograph. The introduction of ART among HIV–infected individuals co-infected with TB could be one of the most important factors in modifying the clinical presentation of TB disease in HIV infected individuals. Advanced HIV disease is known to be associated with lower rates of sputum smear positivity and therefore less infectivity [29]. In this new era in which many countries have scaled up ART delivery, ART could result in increased infectiousness of an individual as improved immune status shifts towards sputum smear positivity. With the move towards integrated HIV/TB care, the potential for transmission of *M. tuberculosis* in clinic settings is increased. WHO has recommended implementation of TB infection control measures, especially in healthcare facilities providing HIV care. These measures include a TB infection control plan comprised of managerial (e.g. use of building space, on-going TB surveillance of healthcare workers); administrative (e.g. triage to identify people with TB symptoms, separation of infectious cases, reduction of waiting times); environmental (e.g. maximising ventilation systems) and personal protection measures (e.g. use of N95 particulate respirators by healthcare workers) to reduce nosocomial TB transmission. Individuals living with HIV should be provided with ART and isoniazid preventive therapy if eligible [30].

In the study 25% of eligible study participants had unknown HIV status. This should not affect the comparisons of HIV positive and negative individuals, and since those with unknown status had similar socio-demographic characteristics as those with known status it should not affect generalisability of the results. The high proportion smear positive in this study may be partly attributed to restriction of the study to those who were smear or culture positive, and to examining smears in a research environment using fluorescence microscopy, which may not be readily available under routine health services and which has been shown to be more sensitive than ZN microscopy [19]. CD4 cell counts were not routinely available so we were unable to explore how the immune status affected the smear status. Although some patients self-reported their ART status, the availability of data from health passports and ART registered patients in the district is likely to have minimised misclassification.

Only 310/766 (40%) of the pulmonary TB cases had chest radiographs. Some of the patients probably had missing radiographs because they had died before a radiograph could be taken. Since chest radiographs were mainly done in individuals eligible for immunological studies, which excluded pregnant women and individuals taking steroids or suffering from diabetes or cancer, this could have affected generalisability of the results, but only 14 (1.8%) of the laboratory-confirmed pulmonary TB patients with known HIV status were excluded from immunological studies based on these criteria. Although there were some differences in gender and HIV prevalence between those who had a radiograph and those who did not, there was little difference by age, socio-economic status or clinical presentation.

The sample size for this analysis was small so there was limited power to investigate effects by duration of ART. Some patients might have had active subclinical disease at the time of ART initiation and presentation of symptomatic disease might have arisen from ART-induced restoration of an immune reaction against *M. tuberculosis* (unmasking) [21]. There is a suggestion that cavitation was more common later, which would be expected with further improvement in immune function [27].

## Conclusion

Wide scaling up of ART in developing countries and early initiation of treatment is likely to change the clinical presentation among TB patients with HIV co-infection to a pattern more similar to that in HIV negative TB patients. While this is more easily diagnosed, especially in settings relying on sputum microscopy, it is also more infectious than the disease in HIV co-infected TB patients who are ART-naive. Infection control measures in ART clinics are important to limit the spread of TB.

## Competing interests

The authors declare no competing interests.

## Authors’ contributions

The study was designed by AC, LM and NF. The study was conducted by LM, FC, MK and NM, with support from AC. The analysis was led by LM, with support from SF, AC, PK and JG. The first draft was written by LM with SF, PK, AC and JG. All authors contributed to revising the manuscript. All authors read and approved the final manuscript.

## Pre-publication history

The pre-publication history for this paper can be accessed here:

http://www.biomedcentral.com/1471-2334/14/107/prepub
